# Modeling Solute Transport by DLA in Soils of Northeastern Egypt

**DOI:** 10.1371/journal.pone.0119943

**Published:** 2015-03-19

**Authors:** Yasser Ahmed Hamed, Hiroshi Yasuda, Magnus Persson, Ronny Berndtsson, Xin-ping Wang

**Affiliations:** 1 Civil Engineering Department, Faculty of Engineering, Port Said University, Port Fouad Port Said, Egypt; 2 Arid Land Research Center, Tottori University, Hamasaka Tottori, Japan; 3 Department of Water Resources Engineering, Lund University, Lund, Sweden; 4 Center for Middle Eastern Studies, Lund University, Lund, Sweden; 5 Shapotou Desert Research and Experiment Station, Cold and Arid Regions Environmental and Engineering Research Institute, Chinese Academy of Sciences, Lanzhou, PR China; Dowling College, UNITED STATES

## Abstract

Arid soils in Egypt display large variability in solute transport properties, causing problems in soil management. To characterize this variability, dye infiltration experiments were conducted on four plots representing three main soil types in northeastern Egypt. The plots represented both cultivated and uncultivated land use. The observed dye patterns displayed a large variability and especially the clay soils indicated a high degree of preferential flow. The loamy sand and sandy soils displayed a more uniform dye distribution indicating more homogeneous soil properties. The observed dye patterns were modeled using a diffusion limited aggregation (DLA) model. The DLA is a random walk model where model parameters can be optimized using genetic algorithms (GA). The DLA model reproduced the observed dye patterns for all soils in an excellent way. The best fit was obtained with a specific combination of directional random walk probabilities P_u_, P_d_, P_r_, and P_l_ for each plot (correlation 0.97–0.99). To account for soil layers with different hydraulic properties a two layer DLA model was developed. For all plots the P_u_ (upward random walk probability) was higher for the upper more homogeneous soil layer. The overall results showed that spatial variability resulting from solute transport for the investigated soils can be modeled using a DLA approach.

## Introduction

Preferential flow, especially for unsaturated field soil conditions, is an important issue in soil science [1]. Not only structured soils but also seemingly homogeneous soils often display preferential flow patterns [2–5]. In most practical engineering problems in soil physics, e.g., irrigation, it is assumed that solute transport is homogeneous [6]. Transport in actual field soils are, however, always more or less heterogeneous. A detailed understanding of the reasons for the heterogeneity is still not fully resolved. Often it is the complicated three-dimensional flow structures at the micro-scale that determine the observed field scale variation [7–8].

Using dye as a tracer is useful to visualize spatial flow patterns and, consequently, it has been widely used by soil scientists. Dye infiltration can show typical percolation patterns for different types of soil. In Tunisia, dye experiments in a clayey soil showed that dye moved along ped faces and distinct cracks [8]. In [2] the authors studied the toxicity of Brilliant Blue FCF. They found that Brilliant Blue FCF is a useful dye tracer to stain the flow paths of water in soil media. The Brilliant Blue FCF can be considered as an environmentally acceptable tracer for studying solute transport in soil, especially at field scale.

A solute transport model was developed based on diffusion-limited aggregation (DLA) by [9]. Using the DLA concept to model transport of solutes in soil proved that complicated three-dimensional patterns can be simulated in a simple way. The DLA model can generate fractal transport patterns often observed in subsurface flow phenomena [10–15]. The DLA model indicates the intricate conductive soil network for infiltrating fluids from a given infiltration occurrence.

[16] used the DLA model to simulate solute concentration at three field plots with different accumulated infiltration per unit time. Since the DLA model is stochastic, each DLA realization gives slightly different results. Thus, it is possible not only to simulate horizontally averaged solute transport in a certain soil volume but also the variability. Consequently, the DLA model can be said to be a data-driven method that follows stochastic concepts. It mimics the complex conducting soil network in up to 3 dimensions. Thus, the model can be used to replicate observed transport patterns in the soil based on a set of few and simple parameters. One of the disadvantages is that it does not elucidate the dynamics of transport patterns since it merely gives the final state of infiltration. A further disadvantage is that it does not give information about concentration changes of the infiltrating solute. A major advantage is, however, its relative simplicity in terms of parameter use and possibility to simulate complex patterns.

[16] showed that a DLA model not only could estimate the horizontal average transport but also the observed standard deviation of dye coverage versus depth. In order to generalize model results further studies are needed to determine parameter variability for different soil types. In view of this, the objective of the study was to investigate and compare three main soil types in northeastern Egypt regarding solute transport properties. The DLA modeling was used for reproducing observed dye patterns for these field soils. The physical meaning of the model parameters and the differences in the parameter values between the different soil types were evaluated and discussed.

## Materials and Methods

### Area description

**Fig 1:**
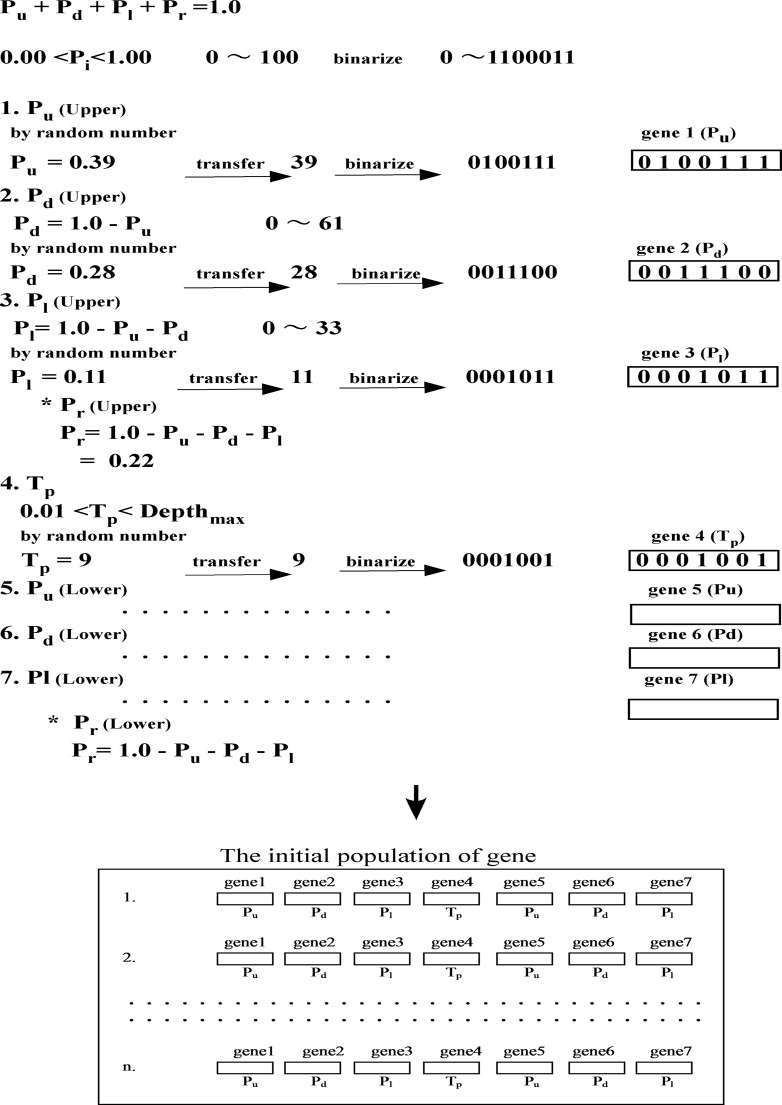
Gene selection process. (Genes corresponding to DLA parameters considering the continuous equation, P_u_ + P_d_ + P_l_ + P_r_ = 1.0, DLA parameters (P_u_, P_d_, P_l_, and P_r_ of the upper layer, T_p_, P_u_, P_d_, P_l_, and P_r_ of the lower layer) were generated by random numbers and transformed to binary gene values (strings). A group of genes forms a population).

The experiments were carried out in October 2004 at four plots (Plot 1–4) located in northeastern Egypt (Fig. 1). The experimental plots were located on private land (for future permissions please contact the first author). For the presented experimental activities no specific permissions were required. Further, the field experiments did not involve endangered or protected species. Two plots were located west of the Suez Canal, 18 km south of Port Said City (Plot 1–2; 31N 01´ 38.18”, 32E 17´ 57.92”). Plot 1 had not been used for any previous agricultural activity and thus represented natural conditions but Plot 2 had been cultivated during four years before the experiments. Plots 3–4 were located at the Sahl El-Tina plain in Sinai east of the Suez Canal, 50 km south of Port Said city (Plot 3; 30N 59´18.18”, 32E 26`7.36”; Plot 4; 30N 59´ 43.61”, 32E 27´ 56.13”). These two plots were used for agriculture during the previous four years. The soil types are clayey soil at the western part of Suez Canal (Plot 1–2), loamy sand (Plot 3), and sandy soil (Plot 4) at the eastern part. The investigated soils represent three main soil types within the El-Salam Canal project area. Soil properties of the plots are described in Table 1. The table shows general differences in soil texture for the different plots. The main difference between the plots is, however, not the texture but instead the soil structure that results in differences in solute transport properties. Plot 1 is uncultivated heavy clay soil with developed shrinkage cracks especially in the vertical. Plot 2 is also heavy clay but a soil that was cultivated during four years previous the experiments. The natural large-scale prismatic clay structure is therefore developing in to smaller peds and aggregates for upper soil layers. Plot 3 is a loamy sand soil with interspersed clayey patches. Finally, Plot 4 is a sandy soil that appears to be rather homogeneous without structure. The organic matter content of Plots 1–2, 3, and 4 is 1–1.3%, 0.4–0.5% and less than 0.1%, respectively. The climate at the time for experiments was dry and warm. The mean temperature was 25°C and there was no rain on any day during the experimental period. The mean humidity was 65%.

**Table 1:** Soil properties of the four plots.

	Depth (cm)	Clay (%)	Silt (%)	Fine Sand (%)	Bulk density (t/m^3^)
**Plot 1**	0–10	71	26	4	1.80
	10–30	67	30	4	1.90
	30–50	68	29	3	1.87
**Plot 2**	0–10	65	31	4	1.77
	10–30	68	28	4	1.87
	30–50	67	31	2	1.73
**Plot 3**	0–10	8	12	80	1.38
	10–30	6	14	80	1.37
	30–50	10	9	81	1.38
	Clayey patches	68	28	4	1.81
**Plot 4**	0–10	4	5	91	1.68
	10–30	4	5	91	1.66
	30–50	4	4	92	1.66

The groundwater table is located at about 1.0 m depth at Plot 1–3 and deeper than 1.5 m at Plot 4. The tillage depth was about 0.30 m for Plot 2 (clay soil) and about 1.0 m for Plot 3–4 (loamy sand soil and sandy soil). More details about the plots can be found in [17].

### Experimental set-up

At each experimental plot, vegetation was carefully removed from the infiltration area (1 m × 1 m) and the surface was gently leveled using hand tools. An iron frame (1 m × 1 m × 0.25 m) was pushed into the soil to about 0.1 m depth. Local irrigation water was used for the infiltration experiments. The irrigation water had an average electrical conductivity, ECP, of 1.7, 3.5 and 3.2 dS/m for Plots 1–2, 3, and 4, respectively. The irrigation water was mixed with 5 g/l food-grade dye pigment Vitasyn-Blau AE 90 (Swedish Hoechst Ltd.). This dye has the same chemical composition as the Brilliant Blue FCF, which has been frequently used in field experiments [18–20]. By using a watering can, manual irrigation, which simulates the flood irrigation in the area, was applied slowly in one dose to irrigate the soil surface corresponding to 14 mm/day of the mixture during three successive days. About 15 mm is typically used in the area as a daily irrigation volume. An area extending 0.25 m in all directions around the plot was irrigated in order to avoid boundary effects. The same procedure of irrigation was used for each plot. Between irrigation events, the plots were covered with a plastic sheet in order to prevent evaporation losses. The fourth day after irrigating with the mixture of water and dye, a trench was dug and eight vertical soil sections were excavated at 0.1 m intervals. The soil sections were excavated in depth until no dye traces were detected. This meant in all cases down to a maximum depth of 0.5 m.

### Image analysis

Vertical soil sections were photographed with a digital camera from the same horizontal distance for each section (1.5 m). The pictures were taken with a resolution of 2592 × 1944 pixels. The digital images were analyzed using Adobe Photoshop CS. The investigated sections of 100 × 50 cm were scaled to 2000 × 1000 pixels. Consequently, a resolution of 0.5 mm in both horizontal and vertical direction was obtained. In order to transfer the color photos into black and white, the images were converted into the CMYK color space. In the Cyan channel, stained and unstained areas clearly appeared as light and dark gray. A threshold gray scale level was selected for each image in order to convert unstained soil to white and stained soil to black. The technique was tested using different thresholds and then routinely checked by comparing the color photos with the black and white pictures by eye so as to minimize errors in this process. The black and white pictures were imported as TIFF files into Mathworks Matlab, where they were analyzed determining horizontally averaged dye coverage and maximum depth of dye penetration.

While the original data had a resolution of 0.5 by 0.5 mm, the data had to be rescaled for the DLA simulations. The primary reason was to increase the computational speed of the DLA simulation. The DLA grid size needs to be larger than the image data since the DLA cluster needs to be converted from micro scale to macro scale using an artificial measurement [9,16]. Also, by rescaling the image data noise is averaged out. In the present study we used a final resolution of 1 by 1 cm per pixel so that the dye images were 100 by 50 pixels.

## Theory

### DLA modeling concept

[21] introduced a DLA model of a so-called line seed type, which is used in the present study since it mimics the surface boundary condition of solute transport in the field. An analytical plane (two dimensional grids) corresponding to the vertical cross-section in the field was set up for the computer programming. The DLA model generates clusters by randomly walking particles. Particles are randomly generated from a line source at a certain depth below the soil surface (top boundary). By random walk the particles finally reach up to the line seed (soil surface). When the particles reach the seed they remain attached at that position. New particles that reach the seed or previously attached particles permanently attach and form a gradually increasing cluster. The line source is set only several grid units below the growing DLA cluster. This considerably shortens the computational time but without affecting the shape of the DLA cluster [9]. As the cluster grows, the line source is gradually shifted downwards keeping several grid units below the lowest of the cluster. The process can be halted once some pre-defined maximum depth is reached by the growing cluster, z_max_. If the DLA cluster is to be compared with experimental results, observed z_max_ has to be used. Consequently, since the model to a major extent is data driven results have to be compared to observations in a calibration procedure. This means that the model has to be run many times during continuous parameter calibration. Typical soil properties such as soil porosity is not included in the model since it does not have a soil description. Instead, parameters are calibrated using observed dye infiltration patterns.

The randomly walking particle can move in four directions depending on probabilities, P_u_, P_d_, P_r_, and P_l_, corresponding to upward, downward, right, and left walk in the two-dimensional grid, respectively. The sum of these four probabilities is one. An isotropic random walk model has a uniform probability distribution: P_u_ = P_d_ = P_r_ = P_l_ = 0.25. Anisotropic conditions can be simulated by modifying these probabilities. For example, increasing the probability for walking in the upward direction compensates the screening effect for diffusing particles, and a homogeneous cluster is formed. On the other hand, increasing probability for walking to the left and right directions will make the cluster to form only a few fingers and, thus, simulate preferential flow. The shape of the fingers will also depend on the value of P_u_ and P_d_. If the downward movement is dominant, no ramification of the finger will be produced. Thus, increasing the P_u_ parameter means that transport patterns in a well-developed homogeneous soil will be simulated. On the other hand, by increasing P_l_ and P_r_ a less developed soil with cracks and inhomogeneities can be simulated.

Layered soils may be simulated by including different walking probabilities for different soil layers. This means that the model domain is divided in to several layers and the model is run independently for each layer.

The DLA model used in this study was coded in Fortran programming language and followed the model design described by [9]. Field soil properties are usually heterogeneous and the DLA parameters (P_u_, P_d_, P_l_, and P_r_) may thus also be expected to be non-uniform in the soil profile. Especially, soil properties for shallow soil depth (0–0.3 m) are often different from those of the deeper layers as tillage depth, organic matter content, and evaporation mainly affects shallow soil layers. According to this, observed dye patterns in our study displayed a discontinuous tendency in the vertical direction. Thus, a two layer DLA was used in the modeling. Since the DLA model depends on random number generation, the DLA pattern has a statistical distribution [16]. In general, a large grid number is better for a higher resolution DLA pattern. However, a large grid number DLA model requires long calculation time. A small grid number, on the other hand, will exhibit larger variations between different simulations. The simulated DLA pattern corresponds to micro scale solute transport. However, the resolution of the final macro scale DLA has to be the same as the observed dye image, in our case a 100 pixel wide domain. To convert the micro scale DLA to macro scale, each 2 by 2 grid in the DLA grid was considered as stained if at least one of the four pixels was part of the DLA cluster [21]. In this study the dye image was given in a 1 cm resolution (100 pixels for 100 cm), consequently the DLA grid was 200 pixels wide in order to arrive at the same resolution for the converted DLA patterns and the dye images.

Dye pattern in a specific soil is a result of soil properties such as texture and structure. Consequently, the calibrated model parameters mimic soil properties. For homogeneous soils it may be expected that parameters display isotropic conditions (P_u_ = P_d_ = P_r_ = P_l_ = 0.25). On the other hand, for heterogeneous soils with preferential flow characteristics in the vertical the model parameters P_u_ and P_d_ will tend to dominate over the other parameters. Consequently, the model parameters mimic soil properties.

### Genetic Algorithm (GA) optimization

Parameters of the DLA model were optimized by use of Genetic Algorithm (GA) technique. The GA was developed for multi-optimization and is also used in hydrological model optimization [22–24]. For the DLA model application the random walk probability parameters, P_u_, P_d_, P_l_, and P_r_ had to be identified. They obey the following condition: P_u_ + P_d_ + P_l_ + P_r_ = 1.0. First, P_u_ was estimated according to: 0 < P_u_ <1.0. Then, the condition for other parameters P_d_, P_l_, and P_r_ are: 0 < P_d_ < 1.0 - P_u_, 0 < P_l_ < 1.0 - P_u_ - P_d_. Under these conditions, genes were operated and optimization was performed, thus: P_r_ = 1.0 - P_u_ - P_d_ - P_l_ = 1.0. For the double layer DLA model, depth of the turning point (T_p;_ dividing depth between the two soil layers) was optimized in addition to the 8 parameters (P_u_, P_d_, P_l_, and P_r_ for the upper and lower layer, respectively). The condition for T_p_ was: 0 < T_*p*_ < Z_max_, where Z_*max*_ is the maximum depth of observed dye pattern. The 7 generated genes corresponding to P_u_, P_d_, and P_l_ for the two layers together with T_p_ were estimated (selection, crossover, and mutation) subject to the above conditions. In genetic algorithms crossover and mutation are the two basic operators. During reproduction, crossover denotes a recombination of genes. The parental genes thus mutate (or changes somewhat) to the offspring.

**Fig 2:**
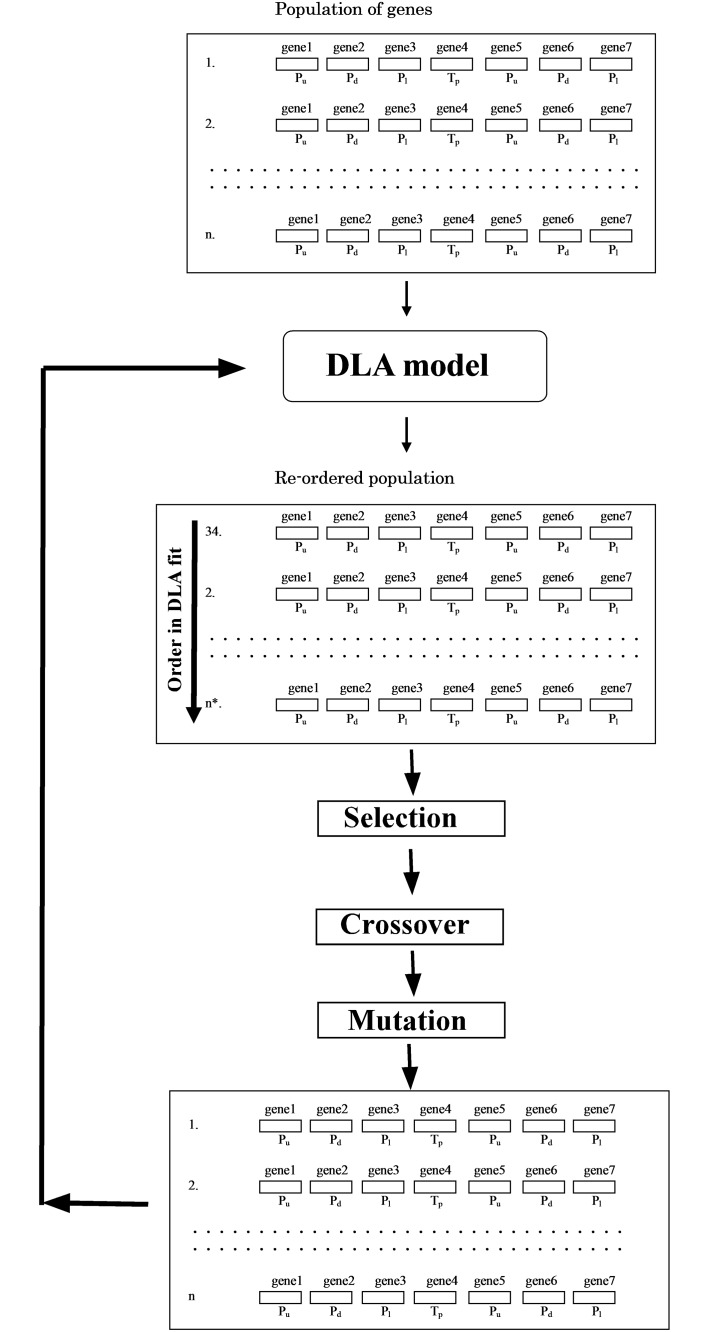
Optimization step of DLA parameters. (The initial population of genes was applied to the DLA model. Combinations of genes were reorganized according to the DLA fit order. Combinations of genes were developed by the selection, crossover, and mutation thus creating a new population).

**Fig 3:**
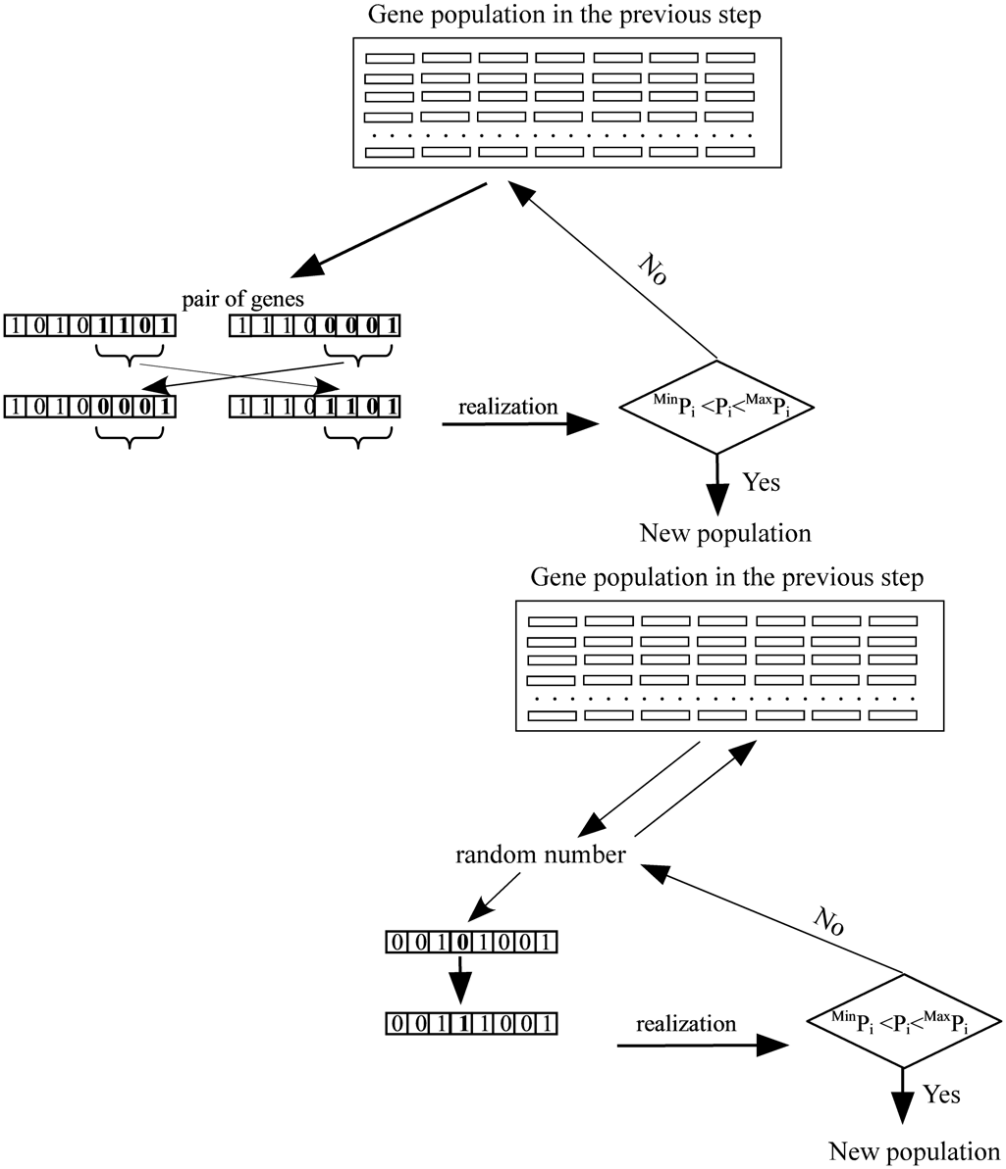
Crossover and mutation process. (A pair of genes was chosen and parts of both genes were replaced. A gene population was the randomly chosen and a part of the binary number was randomly selected for replacement 0 → 1 or 1 → 0).

Considering the continuity equation, P_u_ + P_d_ + P_l_ + P_r_ = 1.0, DLA parameters, P_u_, P_d_, and P_l_ of the upper layer, T_p_, P_u_, P_d_, and P_l_ of the lower layer were generated by random numbers (Fig. 1). Generated parameters were transferred to integer numbers and then transformed to binary values. The binary values (strings) were treated as genes (7 DLA parameters). A combination of the 7 genes was treated as an element in the GA population. Using the random numbers, a population of genes was created and applied to the DLA model parameters (Fig. 2). For the fitting order of the parameters (correlation), combinations of genes were re-ordered. Gene combinations in higher order (higher correlation) were selected as elements of a population for the next step (selection). Pairs of genes in higher correlations were applied to the crossover selection (Fig. 3). Parts of a gene pair were combined for the crossover and a new pair of genes was created. Genes were randomly chosen from the population and a part of each gene was replaced in the mutation process (Fig. 3). For each calculation step, the selection, crossover, and mutation process were applied to the population of parameters to develop genes (Fig. 2). In each step, genes were evaluated to check the range (^*Min*^ *P*_*i*_ <*P*_*i*_< *^Max^* *P*_*i*_). If a parameter was not within the range, another random number was applied or other genes of the population were re-chosen. According to this procedure, population genes were developed and the fit of the DLA parameters was optimized.

## Results and Discussion

### Dye pattern analysis

**Fig 4:**
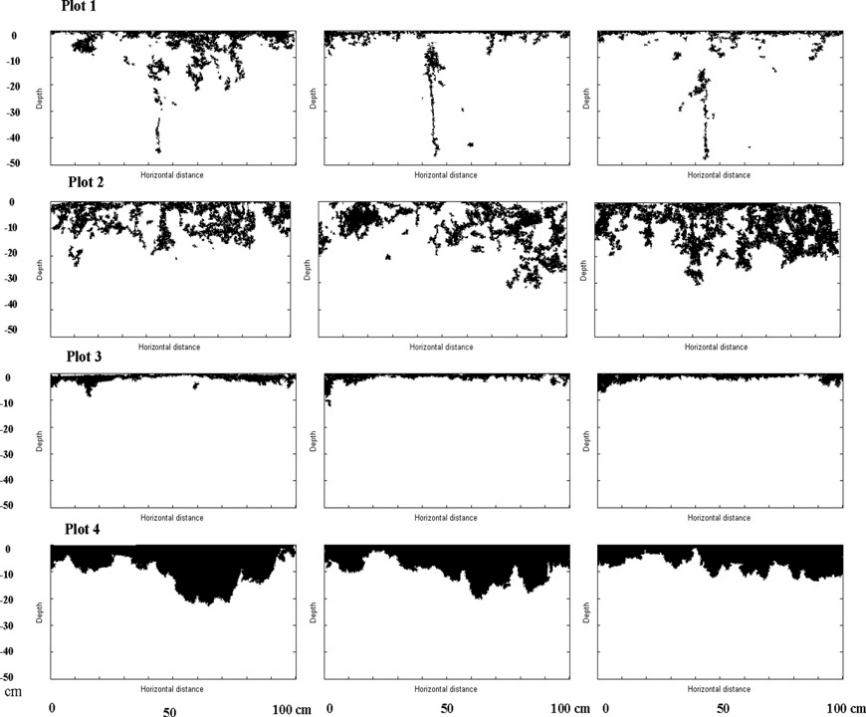
Example of three successive dye pattern sections from each plot [10].

Fig. 4 shows typical black and white images for each plot representing four successive vertical sections. Dye patterns for the remaining vertical sections were similar to those presented in the figure. Fig. 4 shows that strongest heterogeneous dye stained pattern was found in Plots 1 and 2 (natural and cultivated clay soil) as compared to the more homogeneous Plots 3 and 4 (loamy sand and sandy soil). Clayey soils in arid environments usually display strong heterogeneity because of crack formation due to repeated wetting and drying processes. A similar strong heterogeneity of solute transport for clayey soils was reported by [25,8,9]. Recall that the difference between Plots 1 and 2 is that the latter one had been cultivated. Since the top layer is made more homogeneous by agricultural activities such as plowing, cultivating, and seeding, the dye pattern also becomes somewhat more homogeneous. For Plot 1, the average dye infiltration depth was 0.36 m and the maximum depth was 0.47 m.

The dye pattern for Plot 2 indicates flow between and around smaller peds formed by tillage for the upper soil layer. Consequently, peds of different size at the soil surface were often not completely stained by the dye. Also, below the tillage depth (0.3 m), there was only sporadic dye coloring. The average dye depth was 0.27 m with a maximum dye depth of 0.35 m.

Sandy loam (Plot 3) showed rather homogeneous dye distribution for shallow depths. Here, the dye pattern indicated a solute flow that only reached a shallow soil depth. The average and maximum dye depth was only 0.09 and 0.12 m, respectively. This reveals a very limited solute transport in spite of large plowing depth (1.0 m depth).

Sandy soil (Plot 4) displayed a somewhat heterogeneous dye pattern. Here, dye was transported deeper and with rather good coverage of dye. The average and maximum dye depth was 0.19 and 0.23 m, respectively.

**Fig 5:**
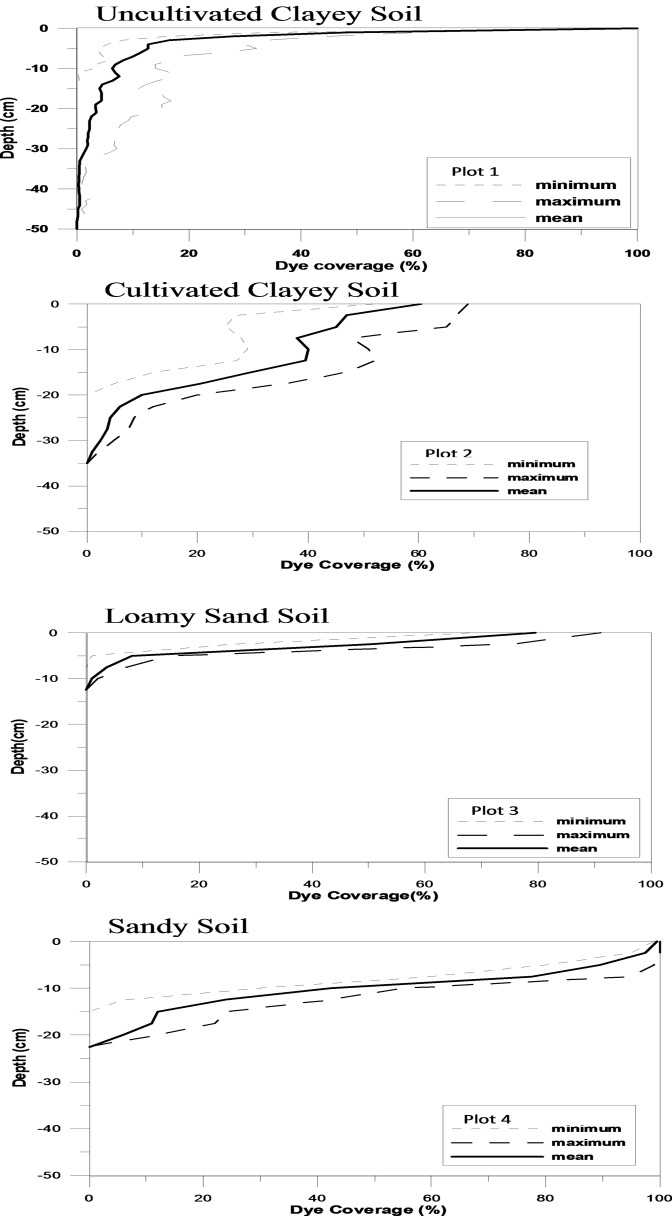
Minimum, maximum, and mean dye coverage for all plots [10].

Fig. 5 shows minimum, mean, and maximum horizontally averaged dye coverage with respect to depth for the eight successive vertical sections for each plot. The horizontal dye coverage indicates the degree of preferential transport in the soil profile. Generally, it decreased rapidly with depth for all plots. In clay soil, average dye coverage decreased from about 65% just below soil surface to 10% at 0.2 m depth. In loamy sand soil the dye coverage decreased rapidly from 80% just below the surface to 10% at about 0.05 m depth. In the sandy soil the dye coverage decreased from 100% at the surface to 10% at 0.17 m depth.

### DLA modeling

**Fig 6:**
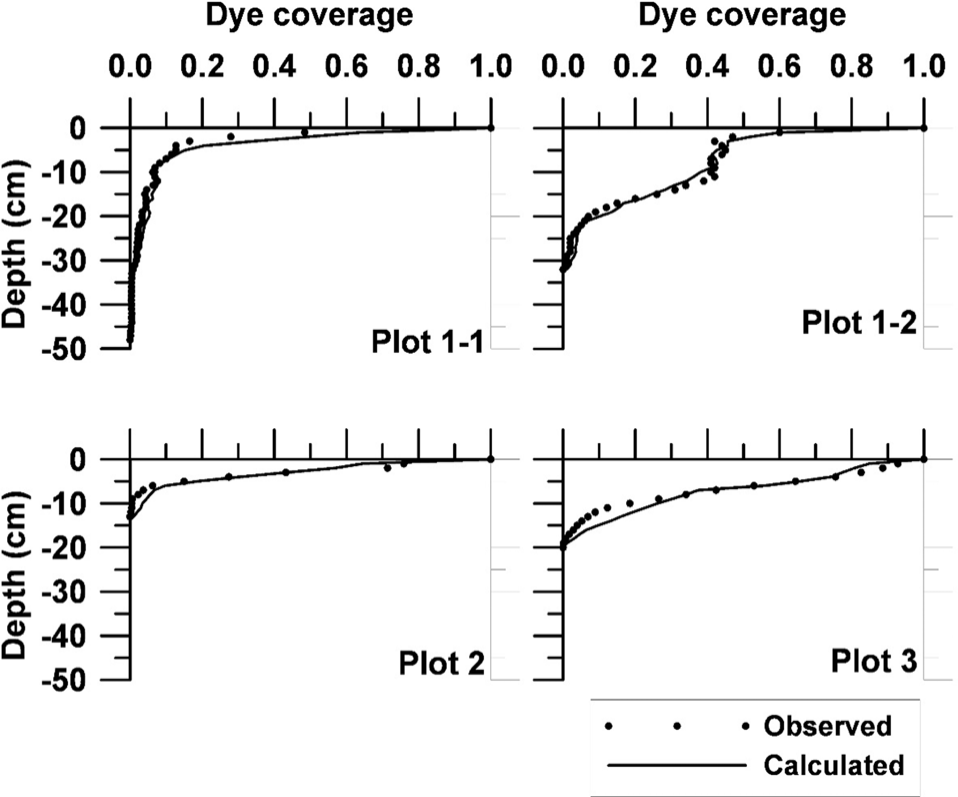
Comparison of between DLA model results and observations. (The horizontal average is given as the mean of 20 realizations).

Since the DLA is a stochastic model, the DLA modeling results have a statistical distribution [16]. Thus, the horizontal average dye coverage distribution with depth was obtained as the mean value of 20 DLA model realizations (Fig. 6). These 20 realizations were used as input to the GA for optimal parameter estimation. The optimized DLA parameters are presented in Table 2. All plots showed a very good fit between the DLA patterns and observed data (r between 0.97–0.99).

**Table 2:** Optimized parameters of DLA for the four plots.

	Optimized parameters								T_P_ (m)	r
	Upper soil layer				Lower layer					
	P_u_	P_d_	P_l_	P_r_	P_u_	P_d_	P_l_	P_r_		
**Plot 1**	0.45	0.27	0.07	0.21	0.32	0.28	0.14	0.26	0.05	0.973
**Plot 2**	0.39	0.28	0.11	0.22	0.28	0.24	0.18	0.30	0.23	0.994
**Plot 3**	0.37	0.20	0.20	0.23	0.33	0.31	0.15	0.21	0.07	0.991
**Plot 4**	0.67	0.12	0.07	0.14	0.33	0.27	0.17	0.23	0.08	0.993

T_p_: dividing depth between upper and lower soil layers

*r*: correlation.

For Plot 1 (natural clay soil with vertical soil cracks), the best fits for the random walk probabilities P_u_, P_d_, P_l_, and P_r_ were 0.45, 0.27, 0.07, and 0.21, respectively, for the upper layer (0–0.05 m) and 0.32, 0.28, 0.14, and 0.26, respectively, for the lower layer (0.05–0.50 m; Fig. 6). This means that the model was run separately for the different layers. As expected, the P_u_ dominates and P_d_ is also large at this site for both depths due to the strong heterogeneity in form of fingering. For both soil layers, particles moved preferably in vertical direction and almost equally downward or upward. As a result, fingered patterns developed in both soil layers (compare Fig. 4).

For Plot 2 (cultivated clay soil), the vertical section was divided into two layers (0–0.23 m and 0.23–0.5 m). The best fits of the random walk probabilities P_u_, P_d_, P_l_, and P_r_ were 0.39, 0.28, 0.22, and 0.22, respectively for the upper layer (0–0.23 m depth) and 0.28, 0.24, 0.18, and 0.30 for the lower layer. Also here, the P_u_ parameter dominates over the other parameters but not as much as for Plot 1. Consequently, the fingering is less developed here even if the heterogeneity still is large. This results in larger contribution from the other parameters P_l_, P_r_, and P_d_. In the upper soil layer, particles moved preferentially more likely upward than downward. Thus, the fingering was more important for the shallower 0–0.23 m soil layer as compared to the deeper 0.23–0.5 m soil layer.

For Plot 3 (loamy sand soil; Fig. 4), the upper layer (0.07 m depth) the parameters were relatively homogeneous (P_u_ = 0.37, P_d_ = 0.20, P_l_ = 0.20, and P_r_ = 0.23). The larger parameter homogeneity and decreased relative probability for walking in the upward direction also decreased development of fingering. For the deeper soil layer, particles moved somewhat more preferentially in vertical direction due to dominating P_u_ and P_d_ (P_u_ = 0.33, P_d_ = 0.31, P_l_ = 0.15, P_r_ = 0.21). Consequently, a tendency for preferential flow along fingers was again generated.

For P4 (sandy soil), the optimum parameters for the upper layer (0–0.05 m) were P_u_ = 0.67, P_d_ = 0.12, P_l_ = 0.07, and P_r_ = 0.14. A dominating P_u_ but small P_d_ means increased probability for walking in the upward direction but with less fingering (P_d_ small influence) but some degree of preferential flow (Fig. 4). For the lower layer (0.05–0.50 m), the walking probabilities changed to P_u_ = 0.33, P_d_ = 0.27, P_l_ = 0.17, and P_r_ = 0.23. Thus, this meant some heterogeneity and some preferential flow (Fig. 4).

The dye pattern of P3 was the least heterogeneous of the investigated soils (loamy sand). The seemingly homogeneous character of the sandy soil (Plot 4) still resulted in heterogeneous solute transport as shown by the dye pattern. Dye patterns for Plots 1–2 (clay soil) displayed a considerable heterogeneity and preferential flow. Especially, the deeper parts at Plots 1–2 showed strong fingering phenomena. [9] also found fingering patterns for large values of P_u_ and P_d_. This corroborates the results of this study. Consequently, it can be said that large P_u_ and P_d_ and small P_r_ and P_l_ indicate strong soil heterogeneity with development of fingered solute transport. As P_d_ decreases also heterogeneity will decrease to some extent. However, a dominant P_u_ will still produce a heterogeneous transport as seen for the sandy soil (Plot 4). More homogeneous DLA parameters with approximately equal size will produce a more homogeneous type of solute transport as seen for the upper layer of the loamy sand soil (Plot 3). According to the above, there is a rather clear soil physical relationship between DLA parameter values and the type of structure of the soil leading to a specific solute transport pattern.

Table 2 indicates that P_u_ is larger in shallow soil layers (mean of 0.47) and smaller in the deeper layers (mean of 0.30). Larger P_u_ corresponds to formation of preferential flow. Thus, P_u_ can be used as a general indication of the degree of preferential flow. If both P_u_ and P_d_ are large this indicates extreme preferential flow and the formation of fingering.

## Conclusions

Four plots in the El-Salam Canal project area in northeastern Egypt were chosen and dye infiltration experiments were conducted. The plots represented three main soil types for the project area. The first two plots were located west of Suez Canal 18 km south of Port Said city. The other two plots were located at Sahl El-Tina area east of Suez Canal. The soil type at the first two plots (Plots 1–2) is clayey soil while it is loamy sand with separate clayey patches at Plot 3 and sandy soil at Plot 4. Irrigation water (14 l per m^2^) mixed with dye (5 g/l) was applied to the plots each day during three successive days. The day after the last irrigation a trench was dug and a total of 8 vertical 0.1 m thick soil sections were excavated for each plot. Each section was photographed and the dye patterns were recorded.

The DLA model was used to simulate dye penetration observed from infiltration experiments for the four plots. As mentioned above the DLA model mimics the infiltrating solute as observed from dye observations. There is thus no direct description of the soil in the model. Even so, the simple parameters used in the DLA model can be used to distinguish between, e.g., homogeneous and heterogeneous soils.

Two layer DLA was used in this study since soil properties (mainly soil structure) at shallow soil depths were different from those of the deeper layers. Consequently, observed dye patterns showed a discontinuous distribution in the vertical direction. In this study observed dye patterns were given as 100 pixels of digital data for 1 m width. For the analysis, 2 by 2 grid DLA pattern was transferred to macro-scale pattern and compared with observed dye pattern. A DLA grid size of 200 was shown to be sufficient to accurately describe the observed dye data.

Genetic algorithms (GA) were used to calibrate the DLA parameters. For optimal walking probabilities P_u_, P_d_, P_l_, and P_r_ at each plot, the model simulations showed very good agreement with experimental data. By optimizing the walking probabilities, the DLA model could thus successfully simulate dye penetration for different soil types with different land use. The DLA parameters show the walking probability up, down, left, and right (P_u_, P_d_, P_l_, and P_r_) in the two-dimensional model domain. Dominating P_u_ and P_d_ lead to extreme heterogeneity and fingered flow. A dominating P_u_ leads to less heterogeneous solute transport that may be typical for a sandy soil. As the other parameters change and become more equal in size less heterogeneity for observed solute transport is expected. Consequently, the DLA model can be used to simulate dye penetration and corresponding solute transport at sites with similar soil conditions but with no observations.

It should be pointed out that our current DLA model is only calibrated to simulate the horizontally averaged dye coverage. We believe that the information in the DLA cluster generated can provide further insight to transport processes in the unsaturated zone. One possible topic in further studies could be to include information entropy [19] in the calibration process.

## Supporting Information

S1 Dataset (XLS)

S2 Dataset (XLS)

S3 Dataset (XLS)

S4 Dataset (XLS)
